# Investigation of chemical transformations of thiophenylglycoside of muramyl dipeptide on the fumed silica surface using TPD-MS, FTIR spectroscopy and ES IT MS

**DOI:** 10.1186/1556-276X-9-234

**Published:** 2014-05-13

**Authors:** Liana R Azizova, Tetiana V Kulik, Borys B Palianytsia, Aleksandr E Zemlyakov, Viktoriya N Tsikalova, Vasiliy Ya Chirva

**Affiliations:** 1 Chuiko Institute of Surface Chemistry, The National Academy of Sciences of Ukraine, 17 Generala Naumova Str., 17, Kyiv 03164, Ukraine; 2 Taurida National V.I. Vernadsky University, Akademika Vernadskogo av. 4, Simferopol 95007, Ukraine

**Keywords:** Muramyl dipeptide, Temperature-programmed desorption mass spectrometry (TPD-MS), Pyrolysis, Thioglycosides, Electrospray ion trap mass spectrometry (ESI IT MS), Fourier transform infrared spectroscopy (FTIR)

## Abstract

In this study, chemical transformations of benzyl ester of *О*-(phenyl-2-acetamido-2,3-dideoxy-1-thio-β-d-glucopyranoside-3-yl)-d-lactoyl-l-alanyl-d-isoglutamine (SPhMDPOBn) on the fumed silica surface were examined, and the surface complex structure was characterized by temperature-programmed desorption mass spectrometry (TPD-MS), infrared spectroscopy (FTIR) and electrospray ion trap mass spectrometry (ES IT MS). Stages of pyrolysis of SPhMDPOBn in pristine state and on the silica surface have been determined. Probably, hydrogen-bonded complex forms between silanol surface groups and the C = O group of the acetamide moiety NH-(CH_3_)-C = O…H-O-Si≡. The thermal transformations of such hydrogen-bonded complex result in pyrolysis of SPhMDPOBn immobilized on the silica surface under TPD-MS conditions. The shifts ∆*ν* of amide I band (measured from 1,626 to 1,639 cm^−l^ for SPhMDPOBn in pristine state) of 33 and 35 cm^−l^ which occurred when SPhMDPOBn was immobilized on the silica surface may be caused by a weakening of the intramolecular hydrogen bonding of the SPhMDPOBn because the interaction with the silica surface as hydrogen bond with silanol groups is weaker than that in associates.

## Background

It has long been known that non-specific stimulation of the immune system can be brought about by exposure to bacteria or components extracted from bacterial cells [1]. The minimum effective structure responsible for the immunoadjuvant activities of the bacterial cell wall was identified as a sugar-containing peptide of the peptidoglycan component [2,3]. The smallest effective synthetic molecule was found to be an *N*-acetylmuramyl-l-alanyl-d-isoglutamine (MDP) [2,3]. MDP was found to exert numerous immunomodulatory activities. However, the administration of MDP into different hosts was always associated with serious toxicity that hampered its use in man [4]. Therefore, in an effort to generate MDP analogues with reduced toxicity and enhanced biological activities, several hundred derivatives were synthesized by chemical modification of the parent molecule [5-8].

Sulfur-containing compounds play an important role in living organisms in energy metabolism (energy production), blood clotting, and synthesis of collagen (the main protein of connective tissue in animals which is the major constituent of bones, fibrous tissues of the skin, hair, and nails) and also participate in enzyme formation. Thioglycosides are less investigated in contrast to *O*-glycosides. It is known that *O*-glycosidase is able to split *O*-glycosides, including of *O*-arylglycosides, in biological systems. Enzymes capable of cleaving the thioglycosidic bond are less common in nature and occur mainly in plants [9,10]. While *O*-glycosidases are ubiquitous, plant myrosinase is the only known *S*-glycosidase [11]. Thioglycosides possess significantly lower susceptibility to enzymatic hydrolysis than the corresponding oxygen glycosides [12]. Also, thioglycosides have gained widespread use in carbohydrate chemistry as inhibitors of *O*-glycosidase and *O*-glycosyltransferase inhibitors [13]. Nevertheless, unlike intensively investigated *O*-glycosides of MDP, *S*-glycosides have received relatively little attention. Currently, only three *S*-alkyl glycosides of MDP, namely, methyl and butyl β-glycosides and hexadecyl *S*-glycoside, have been obtained [8], although 1-thiomuramyl dipeptide itself was found to possess the adjuvant effect close to the action of muramyl dipeptide [8]. For this reason, we synthesized the thioglycosides of MDP.

Fumed silica with controlled particle size, morphology and surface area, along with its chemical, thermal and easy functionalization properties, is suitable for application in adsorption, catalysis, chemical separation, drug delivery and biosensors [14-20]. Silica nanoparticle-MDP thioglycoside complexes' synthesis is a way of structure modification that results in enhanced bioavailability of MDP thioglycosides and prolonged action and simplifies delivery in biological systems [21]. Parfenyuk et al. [21] have demonstrated the possibility of the application of silica nanoparticles for topical delivery of the immunomodulatory drug glucosaminylmuramyl dipeptide (GMDP; the chemically synthesized natural equivalent of peptidoglycan) to the peritoneal macrophages of women with endometriosis. Researchers have shown that the immunomodulatory effect of GMDP can be increased by its immobilization on silica nanoparticles.

The aim of this study was to examine chemical transformations of thiophenylglycoside of MDP with silica surface and to characterize the structure of the adsorbed films on silica by temperature-programmed desorption mass spectrometry (TPD-MS) and Fourier transform infrared spectroscopy (FTIR).

## Methods

### Materials

Powdery fumed silica (pilot plant at the Institute of the Surface Chemistry, Kalush, Ukraine; with a specific surface area of 270 m^2^/g) was used in this work. Fumed silica was previously heated on air for 2 h at 400°С to remove adsorbed organic substances.

**Figure 1:**
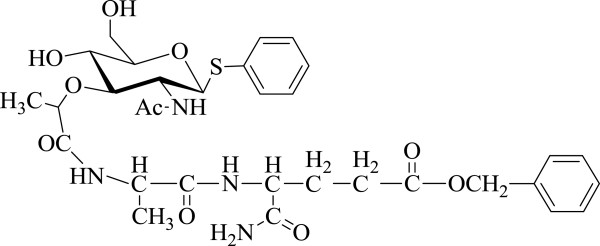
**Structure of *О *-(phenyl-2-acetamido-2,3-dideoxy-1-thio-β-d-glucopyranoside-3-yl)-d-lactoyl-l-alanyl-d-isoglutamine (SPhMDPOBn).**

Benzyl ester of *О*-(phenyl-2-acetamido-2,3-dideoxy-1-thio-β-D-glucopyranoside-3-yl)-D-lactoyl-L-alanyl-D-isoglutamine (SPhMDPOBn; Figure 1) was synthesized at the Department of Biological and Organic Chemistry of Taurida National V.I. Vernadsky University: SPhMDPOBn ^1^H-NMR (DMSO-d_6_) SAr: 7.11 to 7.24 (m, CH_ar_); GlcNac: 4.75 (d, 1 H, *J* = 10 Hz), 1.79 (s, NAc), 7.98 (d, NHAc), 5.58 (d, C4-OH), 4.69 (bt, C_6_-OH); 1.25 (d, CH_3_CHCO); Ala: 1.25 (d, CH_3_), 7.11 to 7.24 (m, NH); Glu: 12.48 (bs, CO_2_R), 2.10 (t, γ-CH_2_), 1.74, 1.95 (m, β-CH_2_), 6.79, 7.24 (s, CONH_2_), 8.28 (d, NH) [22].

The details of the synthesis procedure of SPhMDPOBn have been previously reported [22].

### Loading of MDP arylthioglycosides on the fumed silica surface

The sample of SPhMDPOBn with a concentration of 0.6 mmol/g on the silica surface was obtained by impregnation. It is known that the concentration of free silanol groups (isolated ≡ Si-OH groups), the main active sites, on the silica surface is equal to 0.6 mmol/g of silica [23]. The weight of the MDP thioglycoside batch was such as to ensure a ratio of the concentration of modifier to that of silica surface silanol groups of 1:1. A 0.0121 g of SPhMDPOBn dissolved in 0.8 mL of 96% ethanol was added to 0.03 g of fumed silica in a Petri dish. The components were mixed and left on air at approximately 20°C till the solvent is evaporated (approximately 12 h). In the experiment, the air-dried sample was under investigation.

### Instrument and procedures

#### Electrospray ionization ion trap mass spectrometry analysis

Mass spectra were obtained with the ion trap mass spectrometer Bruker HCT Plus (Bruker Daltonics, Bremen, Germany) equipped with an electrospray ionization source. Ionization was performed under electrospray conditions (flow rate 1.0 μL/min, spray voltage 4.8 kV, sheath gas 40 arb). All spectra were acquired at a capillary temperature of 25°C, and all ion guide voltages were tuned to maximize the abundance of the total ion current.

The analyte solutions (250 pmol/μL) were prepared in methanol. Methanol was of HPLC grade (Sigma, St. Louis, MO, USA).

#### Fourier transform infrared spectroscopy

FTIR spectra were recorded using a FT IR NEXUS spectrometer (Thermo Fisher Scientific Inc., Madison, WI, USA) at room temperature in the frequency range of 4,000 to 400 сm^−1^ in diffuse reflection mode at a resolution of 4 сm^−1^, a scan rate of 0.5 сm/s and number of scans of 150. In diffuse reflectance mode, the powdered samples were mixed with freshly calcined and milled KBr (1:100).

#### Method of temperature-programmed desorption mass spectrometry

TPD-MS experiments were performed in a MKh-7304A monopole mass spectrometer (Electron, Sumy, Ukraine) with electron impact ionization, adapted for thermodesorption measurements. A typical test comprised placing a 20-mg sample on the bottom of a molybdenum-quartz ampoule, evacuating to approximately 5 × 10^−5^ Pa at approximately 20°C and then heating at 0.15°C/s from room temperature to approximately 750°C. For all the samples, the sample vials were filled approximately 1/16 full, which helped limit interparticle diffusion effects [24-28]. Limiting the sample volume along with the high vacuum should further limit readsorption and diffusion resistance as described elsewhere [24-33]. The volatile pyrolysis products was passed through a high-vacuum valve (5.4 mm in diameter, a length of 20 cm and a volume of 12 mL) into the ionization chamber of the mass spectrometer where they were ionized and fragmented by electron impact. After mass separation in the mass analyzer, the ion current due to desorption and pyrolysis was amplified with a VEU-6 secondary-electron multiplier ("Gran" Federal State Unitary Enterprise, Vladikavkaz, Russia). The mass spectra and the *P*-*T* curves (where *P* is the pressure of volatile pyrolysis products, and *T* is the temperature of the samples) were recorded and analyzed using a computer-based data acquisition and processing setup. The mass spectra were recorded within 1 to 210 amu. During each TPD-MS experiment, approximately 240 mass spectra were recorded and averaged. During the thermodesorption experiment, the samples were heated slowly while keeping a high rate of evacuation of the volatile pyrolysis products. The diffusion effects can thus be neglected, and the intensity of the ion current can be considered proportional to the desorption rate.

## Results and discussion

### Electrospray ionization ion trap mass spectrometry analysis of *О*-(phenyl-2-acetamido-2,3-dideoxy-1-thio-β-d-glucopyranoside-3-yl)-d-lactoyl-l-alanyl-d-isoglutamine

**Figure 2:**
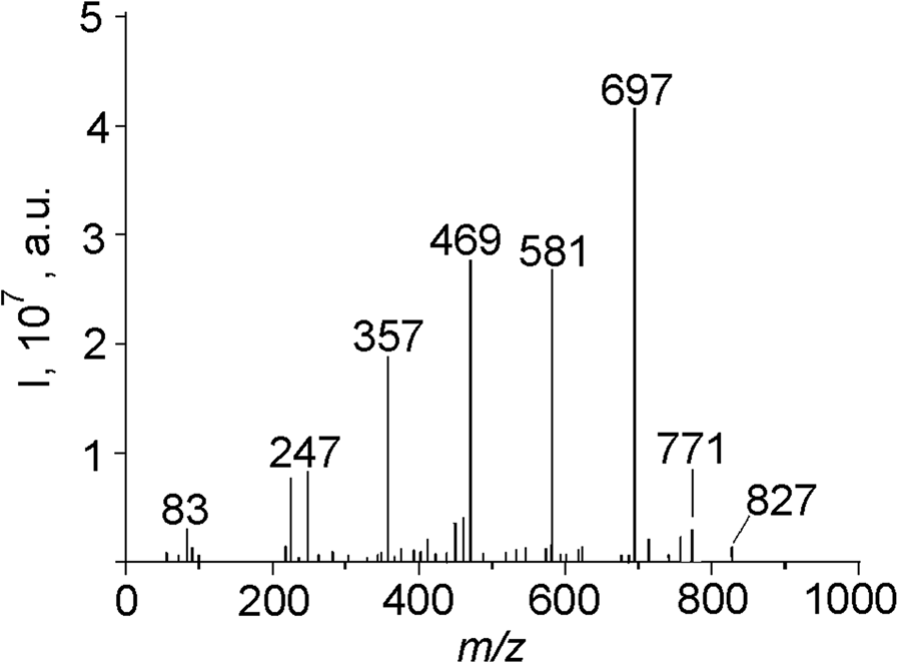
**The positive-mode ESI IT mass spectrum of *О *-(phenyl-2-acetamido-2,3-dideoxy-1-thio-β-d-glucopyranoside-3-yl)-d-lactoyl-l-alanyl-d-isoglutamine (SPhMDPOBn).**

The electrospray ionization ion trap mass spectrum (ESI IT MS) of SPhMDPOBn (Figure 2) shows a peak at *m/z* 697 which corresponds to the sodium adduct molecular ion [M + Na]^+^. The product ion at *m/z* 469 is most probably derived from *m/z* 402 fragment ion of SPhMDPOBn: [M-C_10_H_11_O_2_-C_6_H_5_S + 3Na^+^-2H^+^]^+^. The ion at *m/z* 247 was identified as [M + 3Na]^3+^/3.

### TPD-MS analysis of *О*-(phenyl-2-acetamido-2,3-dideoxy-1-thio-β-d-glucopyranoside-3-yl)-d-lactoyl-l-alanyl-d-isoglutamine

**Figure 3:**
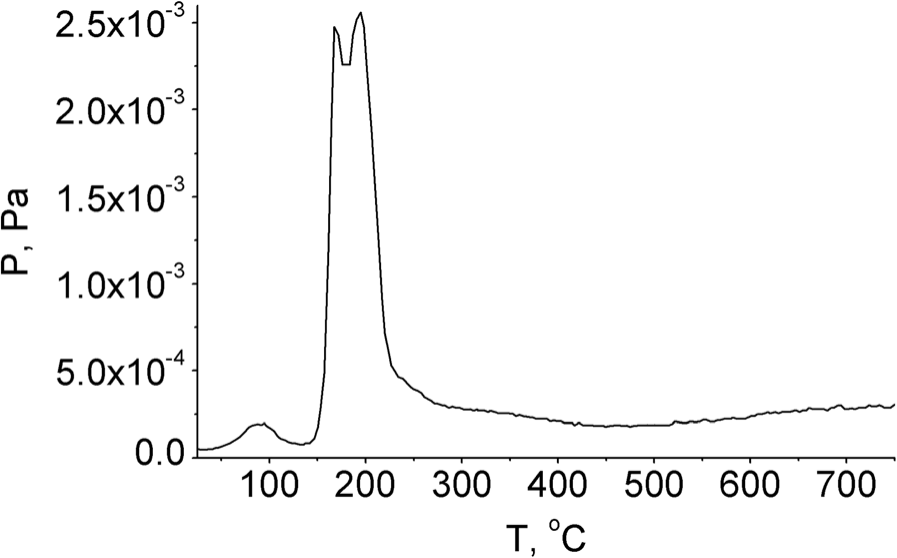
**Temperature-pressure (*P*-*T*) curve of SPhMDPOBn in the pristine state.***P*, pressure of the volatile products; *T*, temperature of SPhMDPOBn.

**Figure 4:**
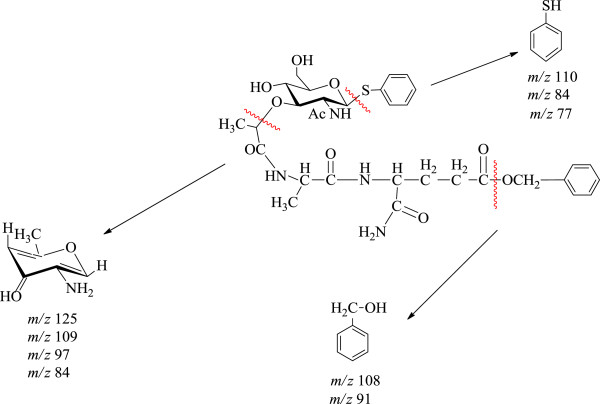
Pyrolysis pattern of SPhMDPOBn under TPD-MS conditions in the pristine state.

**Figure 5:**
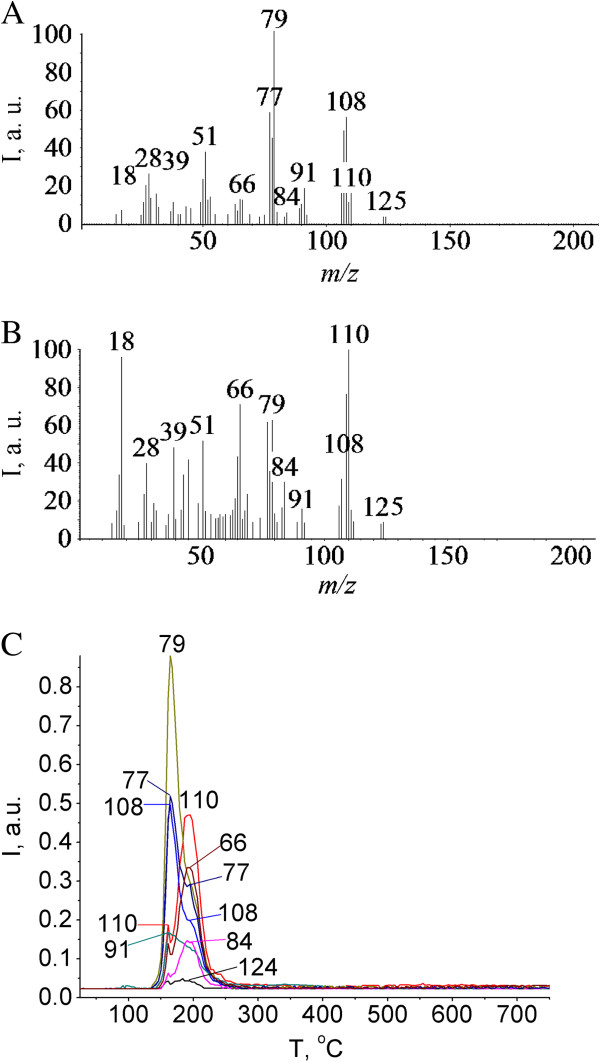
**Pyrolysis of SPhMDPOBn in the pristine state. (A)** Mass spectrum of the pyrolysis products at 163°C, obtained after electron impact ionization. **(B)** Mass spectrum of the pyrolysis products at 194°C, obtained after electron impact ionization. **(C)** Thermograms for *m/z* 124, 110, 108, 91, 84, 79, 77, and 66 under pyrolysis of *О*-(phenyl-2-acetamido-2,3-dideoxy-1-thio-β-d-glucopyranoside-3-yl)-d-lactoyl-l-alanyl-d-isoglutamine (SPhMDPOBn) in the pristine state.

As can be seen from the *P-T* curve (Figure 3), pyrolytic degradation of thiophenylglycoside of MDP in the pristine state proceeds in a relatively narrow temperature range from 150°С to 250°С in two main stages (Figure 4). The same two main stages are observed on the TPD-curves (Figure 5). Probably, these stages of pyrolysis result from the existence of SPhMDPOBn in α- and β-anomeric forms. Figure 4 illustrates a possible pyrolytic pattern and products.

**Figure 6:**
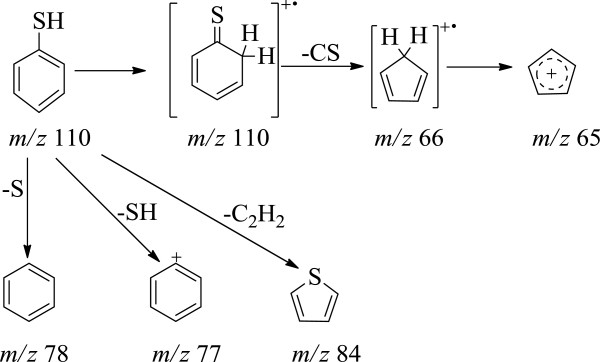
Fragmentation pattern of thiophenol from aglycon under pyrolysis of SPhMDPOBn in the pristine state.

At the first and the second stages (Figure 5), the elimination of the benzyl ester-protected carboxylic group of isoglutamine fragment takes place, which gives rise to a peak of the molecular ion of benzyl alcohol at *m/z* 108 (Figure 4). Fragmentation of benzyl alcohol via loss of the -OH group at *m/z* 17 leads to a common fragment seen for alkyl benzenes at *m/z* 91. Loss of CH_2_OH at *m/z* 31 from the molecular ion gives *m/z* 77 corresponding to the phenyl cation (Figure 4). Loss of aglycone and carbohydrate moiety occurs during the first and the second stages of pyrolysis. But it is observed that there are different ratios of peak intensities on the TPD-curve for molecular and fragment ions of corresponding products. Thus, the first stage proceeds via preferential removal of benzyl alcohol, while the second stage-by elimination of thiophenol. Aglycon is easily removed in the form of thiophenol under the pyrolysis of SPhMDPOBn. The intensity of a thiophenol molecular ion peak is high as the thiophenol molecular ion is stable. The thiophenol molecular ion is stabilized by the presence of π-electron systems, which are capable of accommodating a loss of one electron more easily. The fragmentation of thiophenol molecular ion under electron impact is shown in Figure 6. Isomerization of the molecular ion of thiophenol to thioketone can take place, as the fragmentation pattern used here provides a reasonable explanation of the observed release of -CS and fragment ions from the molecular ion (Figure 6).

**Figure 7:**
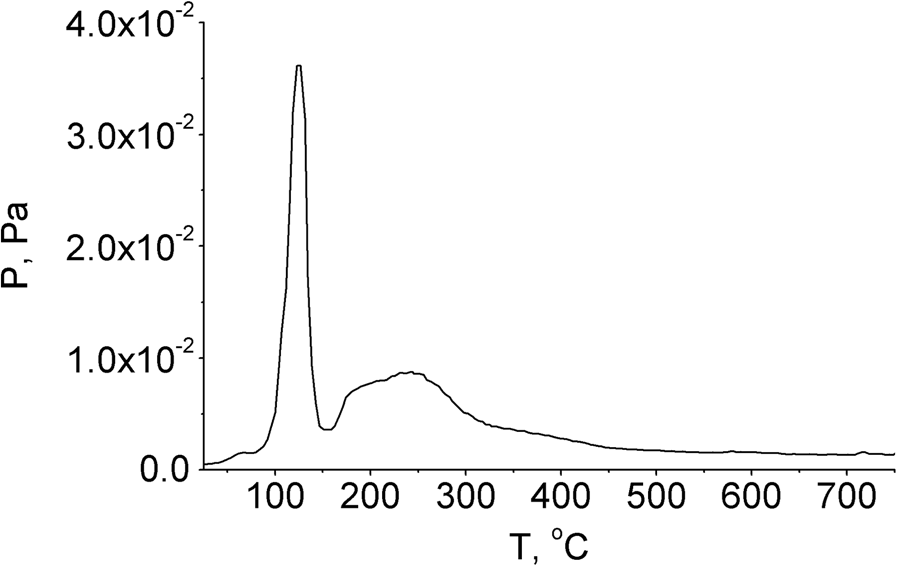
**Temperature-pressure (*P*-*T*) curve of the SPhMDPOBn adsorbed on the silica surface.***P*, pressure of the volatile products; *T*, temperature of the SPhMDPOBn adsorbed on the silica surface.

**Figure 8:**
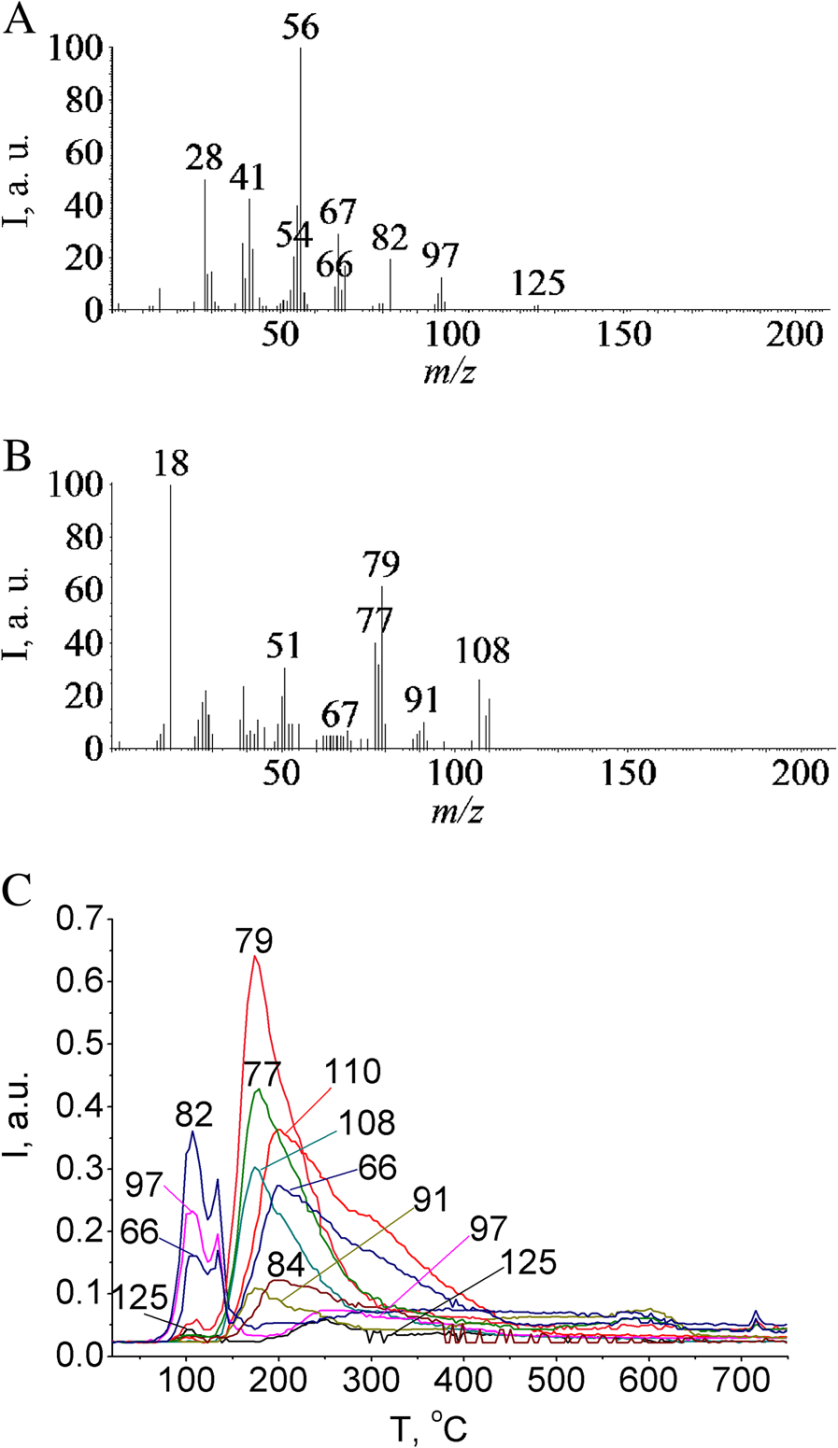
**Pyrolysis of SPhMDPOBn adsorbed on the silica surface (0.6 mmol g**^**−1**^**). (A)** Mass spectrum of pyrolysis products at 105°C, obtained after electron impact ionization. **(B)** Mass spectrum of pyrolysis products at 175°C, obtained after electron impact ionization. **(C)** Thermograms for *m/z* 125, 110, 109, 108, 97, 91, 82, 84, 79, 77, and 66 under pyrolysis of *О*-(phenyl-2-acetamido-2,3-dideoxy-1-thio-β-d-glucopyranoside-3-yl)-d-lactoyl-l-alanyl-d-isoglutamine (SPhMDPOBn) adsorbed on the silica surface.

Moreover, the characteristic peak at *m/z* 125 common to amino sugars is observed in the mass spectrum [34]. Pyrolysis of SPhMDPOBn on the silica surface is more complex. As can be seen from the *P-T* curve (Figure 7), pyrolysis begins at a lower temperature and proceeds in a wider temperature range. At the same time, there are products such as thiophenol, benzyl alcohol and carbohydrate fragment with *m/z* 125, which were observed during the pyrolysis of SPhMDPOBn in the pristine state. However, the sequence of their stages and temperature range are changing. Thermal decomposition of SPhMDPOBn on the silica surface (Figures 7 and 8) also proceeds via the elimination of aglycon and carbohydrate moieties. The set of peaks in mass spectra of SPhMDPOBn adsorbed on the silica surface (Figure 8) is the same as that for the pyrolysis of pristine SPhMDPOBn (Figure 5).

Probably, a hydrogen-bonded complex forms between the silanol surface groups and the C = O group of the acetamide moiety: NH-(CH_3_)-C = O…H-O-Si≡. The thermal transformations of such hydrogen-bonded complex results in the pyrolysis of SPhMDPOBn immobilized on the silica surface under TPD-MS conditions.

### FTIR spectroscopy

**Figure 9:**
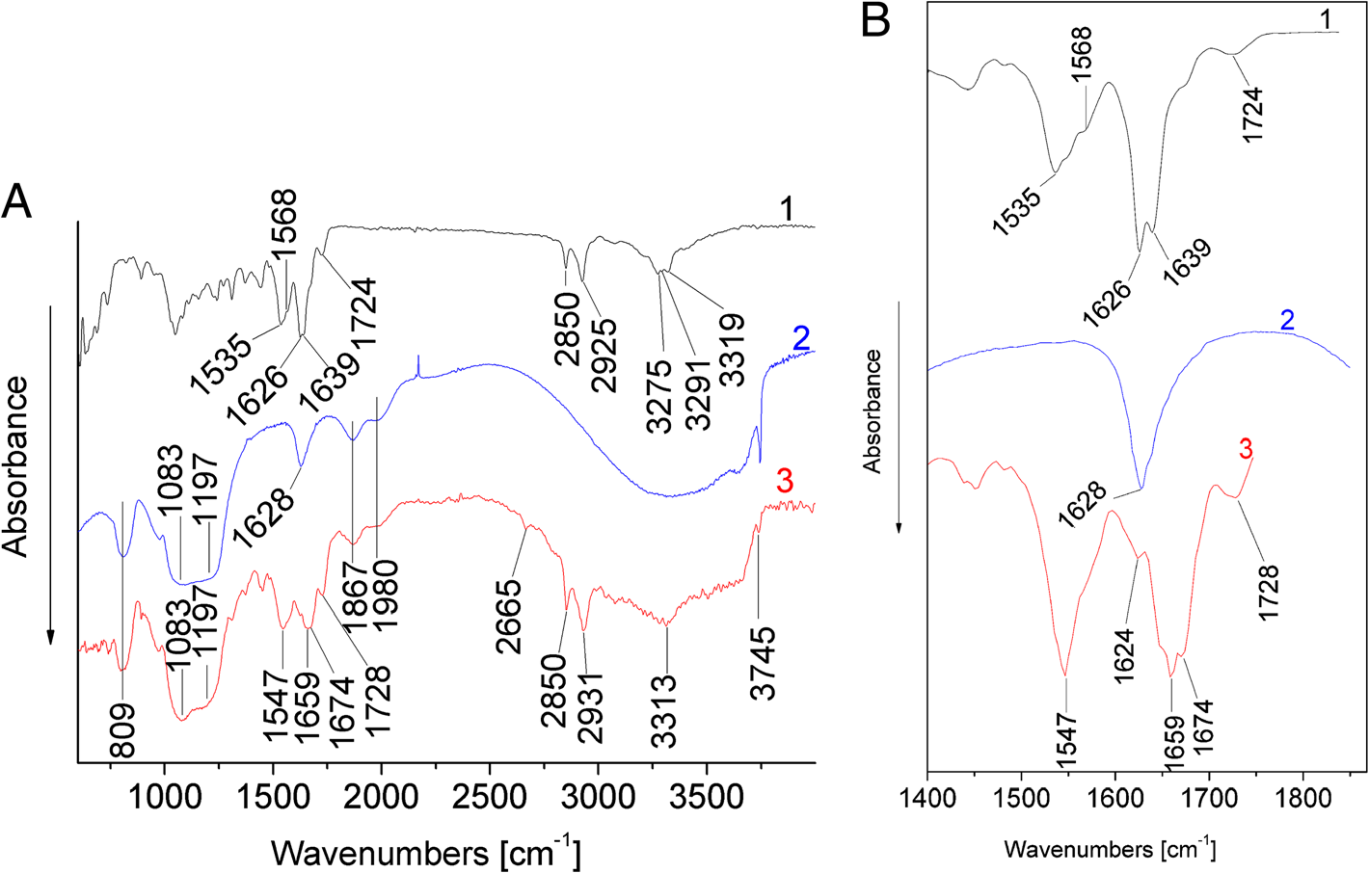
**The IR spectra of the SPhMDPOBn in pristine state and adsorbed on the silica surface. (A)** IR spectra of SPhMDPOBn (line 1), silica (line 2) and silica-supported (impregnated) SPhMDPOBn (0.6 mmol/g^1^, line 3). **(B)** The inset shows the IR bands of SPhMDPOBn (line 1), silica (line 2) and silica-supported (impregnated) SPhMDPOBn (0.6 mmol/g, line 3) in the 1,400- to 1,800-cm^−1^ region from the enlarged spectrum (A).

The IR spectra of the silica sample are depicted in Figure 9. The band at 3,745 cm^−1^ is assigned to the stretching vibration of isolated silanol groups (≡Si-OH). The wide band in the 3,700- to 3,000-cm^−1^ interval corresponds to the overlapping of the O-H-stretching modes of adsorbed water and Si-OH stretchings [35,36]. A small peak at approximately 1,628 cm^−1^ can be attributed to the proton-containing components σ_OH_ (silanol groups and the deformation vibrations of the O-H groups in physically adsorbed molecular water at the silica surface) [37-39]. Bands centered at 1,980 and 1,867 cm^−1^ represent overtones and combinations of intense Si-O fundamental modes (two component bands of Si-O-Si stretching modes) (Table 1).

**Table 1:** **Assignments of the main silica bands in the 700- to 4,000 cm**^**−1 **^**region**

**Band maximum (KBr powder, cm**^**−1**^**)**	**Assignment**^**a**^	**Reference**
3,745	*ν* (isolated silanol groups) Si-OH	[38,40]
3,700 to 3,000	*ν* hydrogen-bonded silanols (overlapping of the stretching modes in hydrogen-bonded hydroxyl bands produced by O-H bonds in adsorbed water and Si-OH)	[38,40]
1,867 and 1,980	Si-O-Si stretching modes	[38,40]
Approximately 1,628 to 1,630	Proton-containing components σ_OH_ (silanol groups and the deformation vibrations of the O-H groups in physically adsorbed molecular water at the silica surface)	[37-39]
Approximately 1,083	Si-O-Si stretching	[38,40]
1,000 to 1,300	*ν*^as^, anti-symmetric stretching of Si-O-Si bonds	[38]
932 to 939	Si-OH stretching	[38,40]
Approximately 809	Bending vibration of Si-O-Si bonds	[38,40]
Approximately 790	Bending modes in Si-OH bonds	[38,40]

^a^ν^as/s^, asymmetric/symmetric stretching mode.

The Si-O-Si and Si-O vibration bands appeared, respectively, at 1,083 and 809 cm^−1^ for the silica sample. The symmetric vibrations of the silicon atoms in a siloxane bond occur at approximately 809 cm^−1^ (ν^as^-Si-O-Si). The largest peak observed in the silica spectrum is present at approximately 1,197 cm^−1^ and is dominated by antisymmetric motion of silicon atoms in siloxane bonds (ν^as^-Si-O-Si).

The infrared spectra of SPhMDPOBn can be divided into several spectral regions. The IR spectra of SPhMDPOBn in the range 4,000 to 3,100 cm^−1^ are dominated by absorption arising from the symmetric and asymmetric N-H stretching modes. The IR spectrum of SPhMDPOBn adsorbed on the silica surface in the range 4,000 to 3,100 cm^−1^ shows a widened band near 3,313 cm^−1^ representing the N-H stretching mode, which is partially overlapped by the bands of the silica matrix (Figure 9). The maximum at 3,313 cm^−1^ is assigned to the N-H groups which were involved in hydrogen bonding interactions with the surface hydroxyl groups.

The bands in the IR spectra of SPhMDPOBn in the pristine state and adsorbed on the silica surface in the region 3,100 to 2,800 cm^−1^ are assigned as the symmetric and antisymmetric stretching vibrations of the С-Н bonds in a methylene group (in pristine state: *ν*^s^ = 2,850 cm^−1^ and *ν*^as^ = 2,925 cm^−1^; on the silica surface: *ν*^s^ = 2,850 cm^−1^ and *ν*^as^ = 2,931 cm^−1^).

The 1,800- to 1,700-cm^−l^ region involves bands due to the C = O stretching modes of benzyl ester-protected carboxylic group of isoglutamine fragment. The bands at 1,724 cm^−l^ in the spectrum of SPhMDPOBn in pristine state and at 1,728 cm^−l^ on the silica surface referred to the ester C = O stretch mode.

The 1,700- to 1,500-cm^−l^ region is dominated by the strong amide I and amide II bands. The conformational-sensitive amide I and amide II bands are the most intensive bands in the spectra of SPhMDPOBn in pristine and adsorbed states. Amide I band absorption originates from the C = O stretching vibration of the amide group, coupled to in-plane N-H bending and C-N stretching modes. The exact frequency of this vibration depends on the nature of the hydrogen bonding involving C = O and N-H groups, which encodes the secondary structure of a dipeptide. The amide I band is usually consists of a number of overlapping component bands representing helices, β-structures, β-turns and random structures. The amide I band of SPhMDPOBn in pristine state consists of two separate component bands at 1,626 and 1,639 сm^−1^ (Figure 9). The amide I band of SPhMDPOBn adsorbed on silica is composed of the following maxima: at 1,659 and 1,674 сm^−1^ (Figure 9, Table 2). The maximum in the spectrum at 1,624 cm^−1^ (Figure 9B, line 3) is assigned to proton-containing components σ_OH_ (silanol groups and the deformation vibrations of the O-H groups in physically adsorbed molecular water at the silica surface). So, amide I and amide II bands are not obscured by overlapping with absorption bands of physically adsorbed molecular water. The intensity of the infrared band at 3,745 cm^−l^ assigned to the OH-stretching vibrations of isolated silanol groups on silica is decreased after immobilization of SPhMDPOBn. This is indicated on the hydrogen bonding of the SPhMDPOBn molecule with silanol groups. The amide I band at 1,626 and 1,639 сm^−1^ was shifted to 1,659 and 1,674 сm^−1^, respectively, for adsorbed-on-silica SPhMDPOBn molecules. That is, the amide I band is shifted to higher wavenumbers (Figure 9, Table 2). The shift of the amide I band of the adsorbed SPhMDPOBn by 33 and 35 cm^−1^, respectively, to higher wavenumbers may be caused by a weakening of the intramolecular hydrogen bonding of the SPhMDPOBn because of the interaction with the silica surface [41,42]. This testifies that the binding to the silica surface occurs due to peptide fragment resulting in the change of its conformation under adsorption. The amide II band represents mainly N-H bending with the C-N stretching vibrations and is conformationally sensitive. The amide II of SPhMDPOBn in pristine state absorbs at 1,535 and 1,568 сm^−1^. The amide II of SPhMDPOBn on the silica surface has a complex structure and centered at 1,547 сm^−1^ (Figure 9, Table 2).

**Table 2:** Absorption frequencies of amide I and amide II bands and N-H stretching modes of SPhMDPOBn

	**Аmide I ( *ν *** **(сm**^**−1**^**))**		**Аmide II ( *ν *** **(сm**^**−1**^**))**		***ν***_**N-H**_**((сm**^**−1**^**))**	
	**Pr**	**Аd**	**Pr**	**Аd**	**Pr**	**Ad**
SPhMDPOBn	1,626	1,659	1,535	1,547	3,275	3,313
	1,639	1,674	1,568		3,291	
					3,319	

Pr, pristine state; Ad, adsorbed on the silica surface.

Earlier using ^1^H-NMR and nuclear Overhauser effect spectroscopy, it was shown that MDP consists of two type II adjacent β-turns forming an S-shaped structure [43,44]. The first β-turn is formed by a C_10_ hydrogen bonding between the Ala NH and the acetamido C = O of *N*-acetylmuramic acid. The existence of the second β-turn is assumed by the presence of a free carboxamide group of isoglutamine [43,44]. Correlation between amide frequency and protein secondary structure found in the literature is listed in Table 2. We can assume from the comparison correlation between amide frequency in FTIR spectra of SPhMDPOBn (Table 2) and protein secondary structure found in the literature (Table 3) that, probably, SPhMDPOBn in the pristine state adopt β-sheet conformation and in the adsorb state, combination of β-sheet and β-turn structures.

**Table 3:** Assignments of amide bands to the secondary structure of peptides and proteins (literature data)

**Assignment**	**Amide I, *ν *** **(сm**^**−1**^**)**	**Amide II, *ν *** **(сm**^**−1**^**)**	**Reference**
α-helix	1,649; 1,653 to 1,657; 1,655	1,545	[45]
	1,648 to 1,660	-	[46]
	1,650 to 1,652	1,540 to 1,546; 1,516	[47]
β-sheet	1,621 to 1,623; 1,630; 1,634 to 1,639; 1,647 to 1,648	1,530	[45]
	1,620 to 1,640; 1,670 to 1,695	-	[46]
	1,633	1,530	[47]
β-turn	1,661; 1,667; 1,673; 1,677	1,528; 1,577	[45]
	1,620 to 1,640; 1,650 to 1,695	-	[46]
	1,663; 1,670; 1,683; 1,688; 1,694		[47]
Random coil	1,648; 1,654; 1,642 to 1,657	-	[45]
	1,640 to 1,657; 1,660 to 1,670	-	[46]

The spectral region 1,400 to 1,200 сm^−1^ is characterized by overlapping deformation vibrations of the C-H bond in methyl and methylene groups of peptide fragment, stretching vibrations of the С-О bond in carbonyl group and amide III vibrations (stretching vibrations of С-N bond and N-H bend in plane) and the Si-O-Si, Si-O and O-Si-O vibration bands of the silica matrix.

## Conclusions

The stages of pyrolysis of aglycone, peptide fragment and carbohydrate residue of thiophenylglycoside of muramyl dipeptide in the pristine state and adsorbed on the silica surface have been determined. Decomposition of thiophenylglycoside of muramyl dipeptide in pristine state occurs within the narrow temperature range from 150°C to 250°C. The decomposition of thiophenylglycoside of muramyl dipeptide adsorbed on the silica surface undergoes certain reactions to produce pyrolysis products such as thiophenol, benzyl alcohol and carbohydrate fragment with *m/z* 125 in the temperature range from 50°C to 450°C. Probably, the hydrogen-bonded complex forms between silanol surface groups and the C = O group of the acetamide moiety NH-(CH_3_)-C = O…H-O-Si≡. The thermal transformations of such hydrogen-bonded complex result in the pyrolysis of SPhMDPOBn immobilized on the silica surface under TPD-MS conditions.

The intensity of the infrared band at 3,745 cm^−l^ assigned to the OH stretching vibrations of isolated silanol groups on silica decreased after the immobilization of SPhMDPOBn. This indicated the hydrogen-bonding of SPhMDPOBn molecule with silanol groups.

The shifts ∆*ν* of the amide I band (measured from 1,626 to 1,639 cm^−l^ for SPhMDPOBn in the pristine state) of 33 and 35 cm^−l^ which occurred when SPhMDPOBn was immobilized on the silica surface may be caused by a weakening of the intramolecular hydrogen bonding of the SPhMDPOBn because the interaction with the silica surface as hydrogen bond with silanol groups is weaker than that in the associates.

## Abbreviations

ESI IT MS: electrospray ion trap mass spectrometry; FTIR: Fourier transform infrared spectroscopy; MDP: muramyl dipeptide; NMR: nuclear magnetic resonance; SPhMDPOBn: *О*-(phenyl-2-acetamido-2,3-dideoxy-1-thio-β-d-glucopyranoside-3-yl}-d-lactoyl-l-alanyl-d-isoglutamine; TPD-MS: temperature-programmed desorption mass spectrometry.

## Competing interests

The authors declare that they have no competing interests.

## Authors’ contributions

LRA obtained the silica-supported SPhMDPOBn sample, carried out the electrospray ionization ion trap mass spectrometric investigation and FT-IR spectroscopic investigation, and drafted the manuscript. LRA together with TVK conceived of the study and participated in its design and interpretation of TPD-MS and FT-IR investigation results. BBP obtained the TPD-MS spectra of SPhMDPOBn in the pristine state and on the silica surface. VNT together with LRA carried out the synthesis of SPhMDPOBn. AEZ together with VYC participated in the design and coordination of the synthesis of SPhMDPOBn. All authors read and approved the final manuscript. We declare that this manuscript is original, has not been published before and is not currently being considered for publication elsewhere.

## Authors' information

LRA is a Ph.D. degree holder and a Junior Research Fellow. TVK is a Ph.D. degree holder, a Senior Researcher, Head of the Laboratory of the Kinetics and Mechanisms of Chemical Transformations on Solid Surfaces. BBP is a Junior Research Fellow. VNT is a Ph.D. degree holder and a Senior Laboratory Assistant. AEZ is a Dr. Sci. holder and a Professor of the Department of Organic and Biological Chemistry, the Faculty of Biology and Chemistry. VYC is Dr. Sci. holder and a Professor and the Head of the Department of Organic and Biological Chemistry, Faculty of Biology and Chemistry.
